# Preclinical evaluation of a novel bilayer collagen nanofiber nerve wrap for accelerating axonal regrowth and myelination in a canine peripheral nerve injury model

**DOI:** 10.1093/rb/rbag193

**Published:** 2026-09-07

**Authors:** Kunkoo Kim, Zhixin Wang, Yaobin Yin, Junlin Chen, Pinyi Lai, Jie Zhang, Zide Wang, Weitao Man, Xiaobin Luo, Yaosai Liu, Yihsien Wu, Haiyan Zhang, Yongjia Zhang, Chun-Yi Yang, Jia Yang, Zheng Cao, Shanlin Chen, Xiumei Wang

**Affiliations:** State Key Laboratory of New Ceramic Materials, School of Materials Science and Engineering, Tsinghua University, Beijing 100084, China; Department of Hand Surgery, Beijing Jishuitan Hospital, Capital Medical University, Beijing 100035, China; Department of Hand Surgery, Beijing Jishuitan Hospital, Capital Medical University, Beijing 100035, China; State Key Laboratory of New Ceramic Materials, School of Materials Science and Engineering, Tsinghua University, Beijing 100084, China; State Key Laboratory of New Ceramic Materials, School of Materials Science and Engineering, Tsinghua University, Beijing 100084, China; Beijing Tsinghua Changgung Hospital, School of Clinical Medicine, Tsinghua University, Beijing 102218, China; Beijing Tsinghua Changgung Hospital, School of Clinical Medicine, Tsinghua University, Beijing 102218, China; Beijing Tsinghua Changgung Hospital, School of Clinical Medicine, Tsinghua University, Beijing 102218, China; Beijing Tsinghua Changgung Hospital, School of Clinical Medicine, Tsinghua University, Beijing 102218, China; State Key Laboratory of New Ceramic Materials, School of Materials Science and Engineering, Tsinghua University, Beijing 100084, China; Department of Neurosurgery, Qilu Hospital (Qingdao), Cheeloo College of Medicine, Shandong University, Shandong 266035, China; Zhejiang Regenbiom Medical Technology Co., Ltd., Tongxiang, Zhejiang 314501, China; Zhejiang Regenbiom Medical Technology Co., Ltd., Tongxiang, Zhejiang 314501, China; Zhejiang Regenbiom Medical Technology Co., Ltd., Tongxiang, Zhejiang 314501, China; Zhejiang Regenbiom Medical Technology Co., Ltd., Tongxiang, Zhejiang 314501, China; State Key Laboratory of New Ceramic Materials, School of Materials Science and Engineering, Tsinghua University, Beijing 100084, China; State Key Laboratory of New Ceramic Materials, Beijing Tsinghua Institute for Frontier Interdisciplinary Innovation, Beijing 102202, China; State Key Laboratory of New Ceramic Materials, School of Materials Science and Engineering, Tsinghua University, Beijing 100084, China; State Key Laboratory of New Ceramic Materials, Beijing Tsinghua Institute for Frontier Interdisciplinary Innovation, Beijing 102202, China; Department of Hand Surgery, Beijing Jishuitan Hospital, Capital Medical University, Beijing 100035, China; State Key Laboratory of New Ceramic Materials, School of Materials Science and Engineering, Tsinghua University, Beijing 100084, China

**Keywords:** peripheral nerve regeneration, bilayer collagen nerve wrap, aligned nanofibers, canine neurorrhaphy model

## Abstract

Peripheral nerve injury remains a major clinical challenge due to unsatisfactory functional recovery after severe nerve damage. Although nerve wraps have emerged as a promising strategy for clinical peripheral nerve repair, conventional nerve wraps still exhibit insufficient topographical guidance during regeneration. In this study, we developed a bilayer collagen nanofiber nerve wrap via blow-spinning technology, consisting of an outer random fibrous layer for structural support and an inner aligned nanofibrous layer for longitudinal neural guidance. To further evaluate its regenerative efficacy and translational potential, a canine neurorrhaphy-assisted peripheral nerve repair model was established. The bilayer nanofiber wraps accelerated organized neural tissue remodeling, aligned axonal regeneration, Schwann-cell-associated remodeling and progressive remyelination throughout the regenerative process. Electrophysiological assessment further demonstrated favorable motor nerve conduction recovery, accompanied by improved locomotor restoration and muscle reinnervation. Importantly, the regenerative performance of the bilayer nanofiber wraps was comparable to, and partially superior to, that of a commercially available sponge-like nerve wrap, particularly in promoting oriented neural regeneration and accelerating myelination. These findings highlight the advantages of aligned topographical guidance in promoting organized peripheral nerve regeneration and support the translational potential of the bilayer collagen nerve wrap as a clinically applicable platform for peripheral nerve repair.

## Graphical abstract

**Graphical Abstract:**
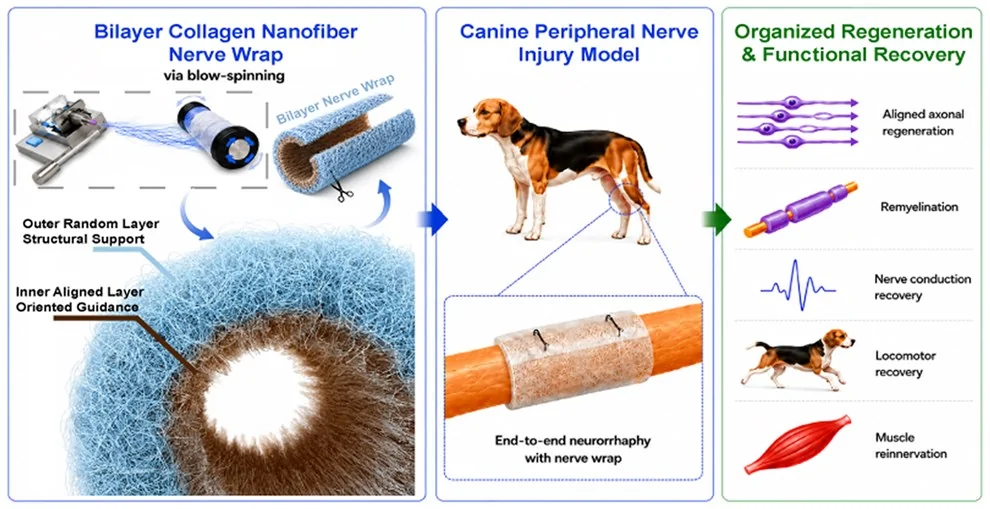

## Introduction

Peripheral nerve injury (PNI) remains a major clinical challenge that frequently results in persistent sensory and motor dysfunction [1–3]. Although the peripheral nervous system possesses a certain intrinsic regenerative capacity, functional recovery following severe nerve injury is often incomplete due to disorganized axonal regeneration [4]. After nerve transection, regenerating axons must successfully traverse the repair interface, re-establish distal target connections and ultimately achieve functional reinnervation of denervated tissues. However, this regenerative process is frequently accompanied by axonal misdirection, aberrant reinnervation, impaired electrophysiological conduction and progressive target muscle atrophy. Microsurgical neurorrhaphy is currently the fundamental and most widely utilized surgical strategy for peripheral nerve repair, serving as the essential basis for direct nerve coaptation, nerve grafting and various reconstructive procedures [5, 6]. Even in autologous nerve grafting, which remains the clinical gold standard for peripheral nerve reconstruction, successful functional recovery still critically depends on effective axonal regeneration across neurorrhaphy interfaces. Despite continuous advances in microsurgical techniques, repaired nerves often fail to fully recover their native structural organization and functional conduction capacity. Conventional end-to-end neurorrhaphy alone still faces several limitations during post-neurorrhaphy regeneration, including insufficient structural support at the coaptation site, perineural soft tissue adhesion and axonal escape from the repair interface, all of which may impair organized axonal regeneration and functional recovery [7]. Therefore, improving the quality and spatial organization of post-neurorrhaphy nerve regeneration remains a critical challenge in peripheral nerve repair.

Biomaterial-assisted nerve wraps have emerged as promising strategies for peripheral nerve repair owing to their favorable biocompatibility, surgical operability and regenerative support capability [8–13]. Compared with conventional nerve guidance conduits, nerve wraps may provide greater surgical flexibility and better adaptability for neurorrhaphy-assisted repair as well as for nerve compression scenarios without complete defect bridging. By surrounding the neurorrhaphy interface, nerve wraps can provide a relatively protected environment, reduce external soft tissue invasion and support nerve healing following microsurgical repair. Among various biomaterials, collagen-based nerve wraps have attracted extensive attention due to their excellent biodegradability, low immunogenicity and intrinsic extracellular matrix-mimicking characteristics [14–16]. Importantly, several collagen-based products have already achieved clinical translation and are widely utilized in peripheral nerve repair, highlighting their considerable translational feasibility and clinical applicability [17, 18]. However, currently available commercial collagen wraps mainly function as passive protective sleeves, while their internal architectures are typically isotropic and randomly porous, providing limited topographical guidance for regenerating axons. Developing structurally optimized nerve wraps capable of guiding organized nerve regeneration may represent an effective strategy for improving post-neurorrhaphy repair outcomes.

Native peripheral nerves are characterized by highly anisotropic and hierarchically aligned architectures, including longitudinally arranged axonal bundles, myelinated nerve fibers and extracellular matrix components [19–21]. These ordered structures collectively support directional electrical signal transmission, coordinated axon–Schwann cell interactions and functional target innervation. Therefore, effective peripheral nerve regeneration requires not only axonal regrowth, but also the reconstruction of a spatially organized regenerative pathway that resembles native nerve architecture. Increasing evidence has demonstrated that the structural organization of regenerative microenvironments plays a critical role in determining the quality of peripheral nerve repair. In this context, biomaterial-based aligned topographical structures provide an attractive strategy for guiding regenerating axons and supporting organized neural remodeling. Compared with randomly distributed structures, aligned architectures can offer directional physical cues through contact-guidance effects, thereby facilitating axonal extension, Schwann cell migration, myelin organization and functional reinnervation. In particular, aligned nanofibrillar architectures possess unique advantages in mimicking the nanoscale structural features of native peripheral nerves, making them promising platforms for biomimetic peripheral nerve repair [22–24].

Based on this concept, we previously developed a bilayer collagen nanofiber conduit using a proprietary blow-spinning fabrication strategy [25]. The conduit consisted of an outer randomly distributed nanofibrous layer that provided favorable structural support and flexibility, together with an inner aligned nanofibrous layer capable of providing directional topographical guidance for regenerating nerve tissues. This bilayer architecture enabled the generation of highly oriented collagen nanofibers that mimicked the aligned structure of native peripheral nerves. In our previous studies using rat peripheral nerve repair models, the bilayer conduit promoted axonal regeneration, remyelination and functional recovery [16]. Building upon these encouraging findings, we further established a scalable and systematic blow-spinning fabrication platform and successfully translated this bilayer collagen nanofiber design into a nerve wrap product for peripheral nerve repair. Nevertheless, despite the promising outcomes observed in small-animal models, the regenerative performance and clinical translational potential of the bilayer collagen nerve wrap in clinically relevant large-animal peripheral nerve repair models remain insufficiently investigated. Therefore, further preclinical validation in large-animal models is essential for evaluating its clinical applicability and translational value [26, 27].

In this study, we further investigated the regenerative efficacy and translational potential of the bilayer collagen nerve wrap in a clinically relevant canine sciatic nerve neurorrhaphy model. Compared with a commercially available collagen product, the bilayer nerve wrap exhibited a biomimetic aligned nanofibrillar architecture that more closely resembled the structural organization of native peripheral nerve tissue. Following sciatic nerve transection and microsurgical neurorrhaphy, the nerve wrap was applied around the repair interface to provide aligned structural guidance during post-neurorrhaphy regeneration. The regenerative outcomes were systematically evaluated through histological analysis, remyelination assessment, electrophysiological examination, locomotor functional evaluation and muscle reinnervation analysis. This study aimed to systematically evaluate whether aligned topographical guidance could promote organized nerve regeneration and functional recovery following neurorrhaphy in a clinically relevant large-animal model. Our findings further highlight the importance of aligned structural guidance in post-neurorrhaphy peripheral nerve regeneration and support the clinical translation of bilayer collagen nerve wraps for peripheral nerve repair.

## Materials and methods

### Fabrication of the bilayer collagen nanofiber nerve wrap

The bilayer collagen nerve wrap used in this study was provided by Regenbiom Medical Technology Co., Ltd. (China), and was fabricated based on our previously reported blow-spinning strategy with minor modifications [25]. The collagen solution (0.2 g mL^−1^) was prepared by dissolving 4 g of type I collagen derived from bovine tendon (Model B, Hebei Junxing Biotechnology Co., Ltd., China) in 20 mL of 15% (v/v) acetic acid (Macklin, China) under continuous stirring for 12 h. The aligned and random collagen nanofibrous membranes were fabricated using a solution blow-spinning system consisting of an airflow source, syringe pump and rotating collector. The detailed blow-spinning parameters were adopted from our previously reported protocol with minor modifications. During fabrication, the collagen solution was continuously extruded through a syringe needle at a constant flow rate of 5 mL h^−1^ under a steady compressed airflow pressure of 0.1 MPa, with a collecting distance of 40 cm between the needle tip and the rotating collector. To fabricate the aligned nanofibrous membrane, fibers were collected on a high-speed rotating mandrel (1200 rpm) to induce aligned fiber orientation. In contrast, random nanofibrous membranes were obtained using a low-speed rotating collector (150 rpm). The thickness of each fibrous layer was controlled by adjusting the spinning duration. The blow-spinning process was carried out under ambient laboratory conditions. The obtained nanofibrous membranes were dried under ambient conditions before wrap assembly. For the fabrication of the bilayer nanofiber nerve wrap, the aligned nanofibrous membrane was first rolled around a cylindrical mandrel to form the inner guidance layer, followed by wrapping with the random nanofibrous membrane to construct the outer protective layer. The resulting bilayer nanofiber nerve conduit was chemically crosslinked using 20 mM 1-ethyl-3-(3-dimethylaminopropyl) carbodiimide hydrochloride (EDC-HCl, Macklin, China) dissolved in 90% ethanol for 24 h at room temperature, followed by extensive washing with ethanol and deionized water prior to vacuum drying. The fabricated bilayer nanofiber conduit was subsequently trimmed into 4 × 20 mm wraps for animal implantation experiments.

Commercially available sponge-like collagen nerve wrap (4 × 20 mm; Tianxinfu Medical Appliance Co., Ltd., Beijing, China) was used as a control product according to the manufacturer’s instructions.

Representative photographs of the nerve wraps under different handling and loading conditions were acquired to qualitatively evaluate structural integrity and surgical handling capability. For mechanical characterization, the tensile and compressive mechanical properties of the bilayer nanofiber nerve wrap were evaluated using a universal testing machine (AG-IC, Shimadzu, Japan). Representative stress–strain curves were obtained, and the corresponding tensile and compressive moduli were determined from the linear region of the stress–strain curves. Three independent samples were tested for each measurement (*n* = 3).

### Structural characterization of the bilayer collagen nanofiber nerve wrap

The surface and cross-sectional morphologies of the commercial sponge nerve wrap and bilayer nanofiber wrap were characterized using field-emission scanning electron microscopy (SEM, Carl Zeiss, Oberkochen, Germany) as previously described [28]. To observe the internal microstructure, the wraps were carefully sectioned to expose the cross-sectional surface. Before SEM observation, all samples were sputter-coated with a 10 nm platinum layer. The surface morphologies of the commercial sponge nerve wrap, outer random fibrous layer and inner aligned fibrous layer of the bilayer nanofiber nerve wrap were observed under different magnifications. In addition, cross-sectional SEM images were obtained to evaluate the hierarchical bilayer architecture and structural integration of the wrap wall.

Fiber orientation distribution analysis of the aligned inner fibrous layer was quantitatively evaluated using ImageJ software (NIH, USA) based on SEM images. The orientation index was calculated as the percentage of fibers distributed within ±10° of the principal alignment direction. Fiber diameter distributions were determined by randomly measuring nanofiber diameters from SEM images using ImageJ software.

### Animal surgery and experimental design

All animal experiments were conducted in accordance with the Guide for the Care and Use of Laboratory Animals and approved by the Animal Care and Use Committee of Beijing Tonghe Litai Biotechnology Co., Ltd. (SYXK (Jing) 2024-0020). A total of 18 adult male Beagle dogs (20 months old, 10–15 kg) were obtained from Beijing Fuhao Experimental Animal Breeding Co., Ltd. (SCXK (Jing) 2022-0004, Beijing, China). Animals were individually housed under standard environmental conditions (20–26°C, 40–70% humidity, 12 h light/dark cycle) with free access to food and water throughout the experimental period.

Before surgery, animals were fasted overnight and anesthetized by intramuscular injection of Zoletil followed by endotracheal intubation and inhalation anesthesia using isoflurane under routine physiological monitoring. After routine sterilization and surgical draping, the sciatic nerve was exposed through a longitudinal incision in the left hindlimb. The surrounding soft tissues were carefully separated under surgical microscopy to fully expose the sciatic nerve trunk. Subsequently, a complete sciatic nerve transection was performed approximately 2 cm above the bifurcation site using microsurgical scissors. After transection, neurorrhaphy was immediately performed under microscopic assistance using 6–0 microsutures for epineurial end-to-end coaptation. Following nerve repair, either the bilayer nanofiber nerve wrap or the commercially available wrap (4 × 20 mm) was wrapped around the neurorrhaphy site and fixed to the surrounding epineurium using interrupted sutures to ensure stable implantation. Representative surgical procedures, including sciatic nerve exposure, transection and wrap-assisted wrapping of the neurorrhaphy site, are shown in Supplementary Figure S1. Animals were randomly assigned into two experimental groups, including the commercial sponge nerve wrap group and the bilayer nanofiber nerve wrap group (*n* = 9 per group). At each postoperative time point (4, 12 and 24 weeks), three animals from each group were used for subsequent evaluations. Histological evaluation, electrophysiological assessment, behavioral analysis and muscle regeneration assessment were performed by investigators blinded to the group allocation. No animals died or developed severe postoperative complications during the experimental period, and no animals or datasets were excluded from the final analyses. During the postoperative period, all animals received routine antibiotic treatment for infection prevention together with daily monitoring of wound healing, food intake, activity and overall physiological condition.

### Hematological and serum biochemical analysis

Peripheral blood samples were collected from all animals at 4, 12 and 24 weeks post-surgery for hematological and serum biochemical evaluation. Whole blood samples were used for routine hematological analysis, including white blood cell (WBC) count, red blood cell (RBC) count, neutrophil (Neu) count and platelet (PLT) count measurements. Serum samples were further analyzed for alanine aminotransferase (ALT), blood urea nitrogen (BUN), creatinine (CRE) and creatine kinase (CK) levels to evaluate potential systemic toxicity and organ-related adverse effects following wrap implantation. All blood analyses were performed according to standard clinical laboratory protocols.

### Histological evaluation

At 4, 12 and 24 weeks post-surgery, animals were deeply anesthetized and euthanized. An approximately 80-mm sciatic nerve segment encompassing the repair site was carefully harvested, and gross photographs of the regenerated nerve specimen were obtained before further processing. The gastrocnemius muscles from the operated and contralateral limbs were also collected, photographed and weighed immediately for muscle recovery evaluation.

For frozen longitudinal section preparation, a 25-mm segment centered on the neurorrhaphy site and encompassing both the proximal and distal regenerated nerve was selected and further fixed in 4% paraformaldehyde and cryoprotected in 30% sucrose solution at 4°C. Subsequently, tissues were embedded in optimal cutting temperature compound and sectioned longitudinally using a cryostat microtome. For hematoxylin and eosin (H&E) staining, frozen longitudinal nerve sections were stained according to standard protocols, and images were acquired using a panoramic slide scanner (3DHIESTECH, Hungary). The orientation distribution of regenerated tissues was analysed using ImageJ software. Immunofluorescence staining was carried out as previously described [29]. The frozen nerve sections were washed with phosphate-buffered saline, permeabilized with 0.3% Triton X-100 and blocked with 10% normal goat serum at room temperature. The sections were then incubated with primary antibodies against NF-200 and S100 overnight at 4°C, followed by incubation with corresponding fluorescent secondary antibodies at room temperature in the dark. Nuclei were stained with DAPI (Abcam, UK). Fluorescence images were captured using a confocal laser scanning microscope under identical acquisition settings. The positive areas of NF-200 and S100 were quantified using ImageJ software. The primary and secondary antibodies are presented in Supplementary Table S1.

For toluidine blue staining and TEM observation, a 4-mm regenerated sciatic nerve segment centered at the neurorrhaphy site was harvested and fixed in 2.5% glutaraldehyde, post-fixed with 1% osmium tetroxide, dehydrated and embedded in epoxy resin. Semi-thin and ultrathin transverse sections were prepared from the central region of the resin-embedded nerve segment. Semi-thin transverse sections were prepared and stained with toluidine blue solution. Images were acquired using a panoramic slide scanner. The density of myelinated fibers was quantified from representative images using ImageJ software. Ultrathin sections of regenerated nerve tissues were prepared from resin-embedded samples for transmission electron microscopy (TEM), further stained with uranyl acetate and lead citrate and observed using a TEM. Myelin sheath thickness, G-ratio and perimeter-based G-ratio were quantified from TEM images using ImageJ software.

For skeletal muscle histology, gastrocnemius muscle tissues were fixed in 4% paraformaldehyde, embedded in paraffin and sectioned transversely. Masson’s trichrome staining was performed according to standard protocols to evaluate muscle fiber morphology and collagen deposition. The muscle fiber area fraction was calculated as the percentage of the analysed tissue area occupied by muscle fibers, whereas the muscle fiber cross-sectional area was determined by measuring the cross-sectional area of individual muscle fibers. The collagen-positive area was quantified as the percentage of collagen-stained area within the analyzed tissue region. All measurements were performed using ImageJ software.

### Electrophysiological assessment

Electrophysiological evaluations were performed at 4, 12 and 24 weeks post-surgery to assess functional neural conduction recovery. Under anesthesia, the regenerated sciatic nerve was surgically exposed, and compound muscle action potentials (CMAPs) were recorded using a neurophysiological recording system (33A07, Dantec Dynamics, Denmark; sampling rate: 48 kHz; band-pass filter: 0.01 Hz–13 kHz). The regenerated sciatic nerve was stimulated at defined proximal and distal sites using supramaximal stimulation, and recording electrodes were inserted into the gastrocnemius muscle. Healthy contralateral nerves were used as references for electrophysiological comparison.

CMAP waveforms were recorded and analysed to evaluate neural signal transmission across the regenerated nerve tissues. Electrophysiological parameters, including CMAP amplitude, latency and motor nerve conduction velocity (MNCV), were quantitatively analysed according to standard electrophysiological protocols. CMAP amplitude was defined as the peak-to-peak voltage of the recorded waveform, whereas latency was defined as the time interval between the stimulus artifact and the onset of the CMAP response. MNCV was calculated by dividing the distance between the proximal and distal stimulation sites by the difference between the proximal and distal onset latencies. All electrophysiological data were processed and quantified under identical experimental settings.

### Behavioral assessment

Behavioral recovery was evaluated at 24 weeks post-surgery through gait observation and motion recording analysis. During assessment, animals were allowed to walk freely in an open observation area while locomotor behaviors were recorded using a digital camera from multiple viewing angles. Representative gait sequences and dynamic locomotor behaviors were extracted from continuous behavioral videos for further analysis.

### Statistical analysis

All quantitative data are presented as mean ± standard deviation (SD). Statistical analyses were performed using SPSS Statistics software (IBM Corp., USA). For each experimental time point, three independent animals were included in each group unless otherwise specified. Comparisons between the commercial sponge nerve wrap group and the bilayer nanofiber nerve wrap group at the same time point were performed using an unpaired two-tailed Student’s *t*-test. Statistical significance was defined as *P *< 0.05.

## Results

### Structural characterization and surgical implementation of the bilayer collagen nerve wrap

To establish a biomimetic nerve wrap capable of simultaneously providing structural support and directional regenerative guidance, a bilayer collagen nanofiber wrap was fabricated using a blow-spinning strategy developed in our previous work. The nerve wrap featured a hierarchically organized architecture composed of an outer random nanofibrous layer and an inner aligned nanofibrous layer. Specifically, the outer layer was designed to provide structural stability and mechanical flexibility, whereas the inner aligned layer was intended to mimic the aligned microarchitecture of native peripheral nerves and facilitate guided axonal regrowth. Commercial collagen sponge wraps with conventional isotropic porous architectures were used as clinically relevant control products throughout the study.

Macroscopic observation further demonstrated distinct structural differences between the commercial sponge wrap and the bilayer nanofiber wrap (Figure 1A). Compared with the sponge-like structure of the commercial wrap, the bilayer nanofiber wrap exhibited a flexible tubular configuration with a well-defined fibrous architecture. In addition, the bilayer nanofiber wrap maintained favorable structural resilience and load-bearing capability under external deformation and loading conditions, indicating suitable mechanical robustness and handling properties for surgical manipulation and neurorrhaphy-assisted implantation (Figure 1B, Supplementary Figure S2 and Video S1). To further quantitatively characterize the mechanical properties of the bilayer nanofiber nerve wrap, tensile and compressive mechanical tests were performed. The nerve wrap exhibited a tensile modulus of 0.43 ± 0.06 MPa and a compressive modulus of 0.60 ± 0.04 MPa, further supporting its favorable mechanical robustness and handling performance for neurorrhaphy-assisted implantation (Supplementary Figure S3).

**Figure 1:**
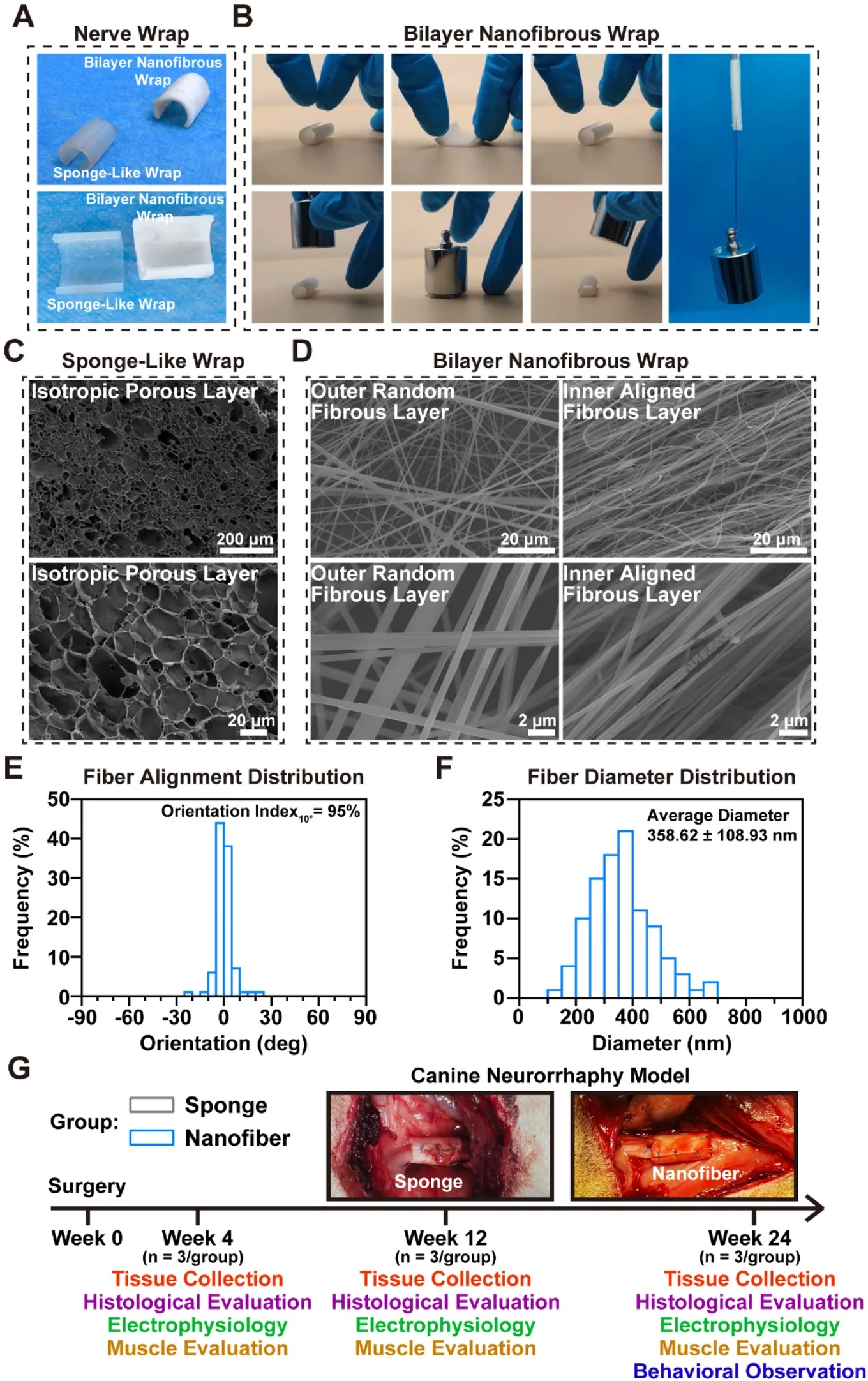
Structural characterization of the bilayer collagen nerve wrap and experimental design for canine peripheral nerve repair. (A) Representative macroscopic photographs of the commercial sponge-like nerve wrap and the bilayer collagen nanofibrous nerve wrap. (B) Representative photographs demonstrating the mechanical flexibility, structural resilience and load-bearing capability of the bilayer collagen nanofibrous wrap under external deformation and loading conditions. (C) SEM images of the commercial sponge-like nerve wrap exhibiting an isotropic porous architecture. (D) SEM images of the outer random nanofibrous layer and inner aligned nanofibrous layer of the bilayer collagen nanofibrous wrap. (E) Fiber alignment distribution of the inner aligned nanofibrous layer. (F) Fiber diameter distribution of the aligned collagen nanofibers. (G) Schematic illustration of the experimental design and canine sciatic neurorrhaphy model. Animals (*n* = 3 per group at each time point) were evaluated at postoperative weeks 4, 12 and 24. Tissue collection, histological evaluation, electrophysiological assessment and muscle evaluation were performed at all three time points, whereas behavioral observation was conducted only at week 24.

SEM further revealed pronounced microstructural differences between the commercial sponge wrap and the bilayer nanofiber wrap. The commercial sponge wrap exhibited a conventional isotropic porous morphology characterized by irregularly interconnected pores distributed throughout the material structure (Figure 1C and Supplementary Figure S4A). In contrast, the bilayer nanofiber wrap exhibited a distinct bilayer fibrous architecture consisting of an outer random layer and an inner highly aligned layer (Figure 1D). Cross-sectional SEM observations further confirmed the successful integration of the bilayer structure, demonstrating a continuous transition from the external random layer to the internal aligned layer (Supplementary Figure S4B). Compared with the porous sponge wrap, the bilayer nanofiber wrap exhibited a denser and more organized nanofibrous microenvironment, which may provide improved structural support together with aligned topographical guidance during nerve regeneration.

To quantitatively characterize the alignment features of the inner fibrous layer, fiber orientation analysis was performed (Figure 1E). The orientation histogram demonstrated that the majority of fibers were distributed within ±10° of the principal axis, yielding an orientation index (OI ± 10°) of approximately 95%, indicative of a highly aligned fibrous topography. In addition, fiber diameter analysis revealed a relatively uniform nanofibrous distribution with an average fiber diameter of 358.62 ± 108.93 nm (Figure 1F), which falls within the characteristic dimensional range of extracellular matrix-mimicking nanofibers. These findings collectively confirmed the successful fabrication of the bilayer nanofiber wrap with well-defined aligned structural features.

A canine sciatic neurorrhaphy model was subsequently established to evaluate the *in vivo* regenerative performance of the nerve wrap systems (Figure 1G). Briefly, following surgical exposure and complete transection of the sciatic nerve, end-to-end neurorrhaphy was immediately performed, after which the coaptation site was wrapped using either a clinically used commercial sponge wrap or the bilayer nanofiber wrap developed in this study (Supplementary Figure S1). Postoperative evaluations were conducted at 4, 12 and 24 weeks, including histological analysis, electrophysiological assessment, behavioral observation and ultrastructural characterization, to comprehensively investigate peripheral nerve regeneration and functional recovery. To further evaluate the systemic biosafety of the implanted nerve wraps, routine hematological and serum biochemical analyses were performed at different postoperative time points. Hematological parameters, including WBCs, Neus, RBCs and PLTs, remained within normal physiological ranges without significant abnormalities between the groups throughout the experimental period (Supplementary Figure S5). Similarly, serum biochemical indicators associated with hepatic, renal and muscle-related functions, including ALT, BUN, CRE and CK, showed no obvious pathological fluctuations following nerve wrap implantation (Supplementary Figure S6). These findings indicate that no evident systemic toxicity was observed in either the commercial sponge wrap or bilayer nanofiber wrap groups during long-term implantation.

### Bilayer nanofiber wrap promoted longitudinally organized nerve tissue regeneration

To comprehensively evaluate regenerative outcomes following neurorrhaphy repair, gross tissue harvesting and histological analyses were performed at 4, 12 and 24 weeks post-surgery. During tissue collection, both the commercial sponge wrap and bilayer nanofiber wrap groups exhibited continuous nerve reconnection across the repair region without obvious rupture, severe inflammatory adhesion or neuroma formation (Supplementary Figure S7). Macroscopic observations further revealed progressive thickening and maturation of regenerated nerve tissues over time in both the groups, indicating stable implantation and successful restoration of nerve continuity.

H&E staining was subsequently performed to evaluate the overall morphology and spatial organization of regenerated nerve tissues. At 4 weeks post-surgery, regenerated tissues in both groups showed newly formed nerve-like structures within the repaired region, indicating the initiation of tissue regeneration following neurorrhaphy repair (Figure 2A). In the commercial sponge wrap group, regenerated tissues appeared relatively loose and heterogeneous, with irregular fibrous organization and dispersed cellular components. In contrast, the bilayer nanofiber wrap group displayed more compact tissue structures with continuous and longitudinally arranged fibrous bundles extending along the repair axis. Correspondingly, orientation histogram analysis revealed that regenerated tissues within the bilayer nanofiber wrap group exhibited a narrower angular distribution concentrated around the principal axis, whereas the commercial sponge wrap group showed a broader and more dispersed orientation profile (Figure 2D and E). These findings suggested that the aligned inner nanofibrous layer of the nerve wrap provided effective topographical guidance for early longitudinal tissue organization during nerve regeneration.

**Figure 2:**
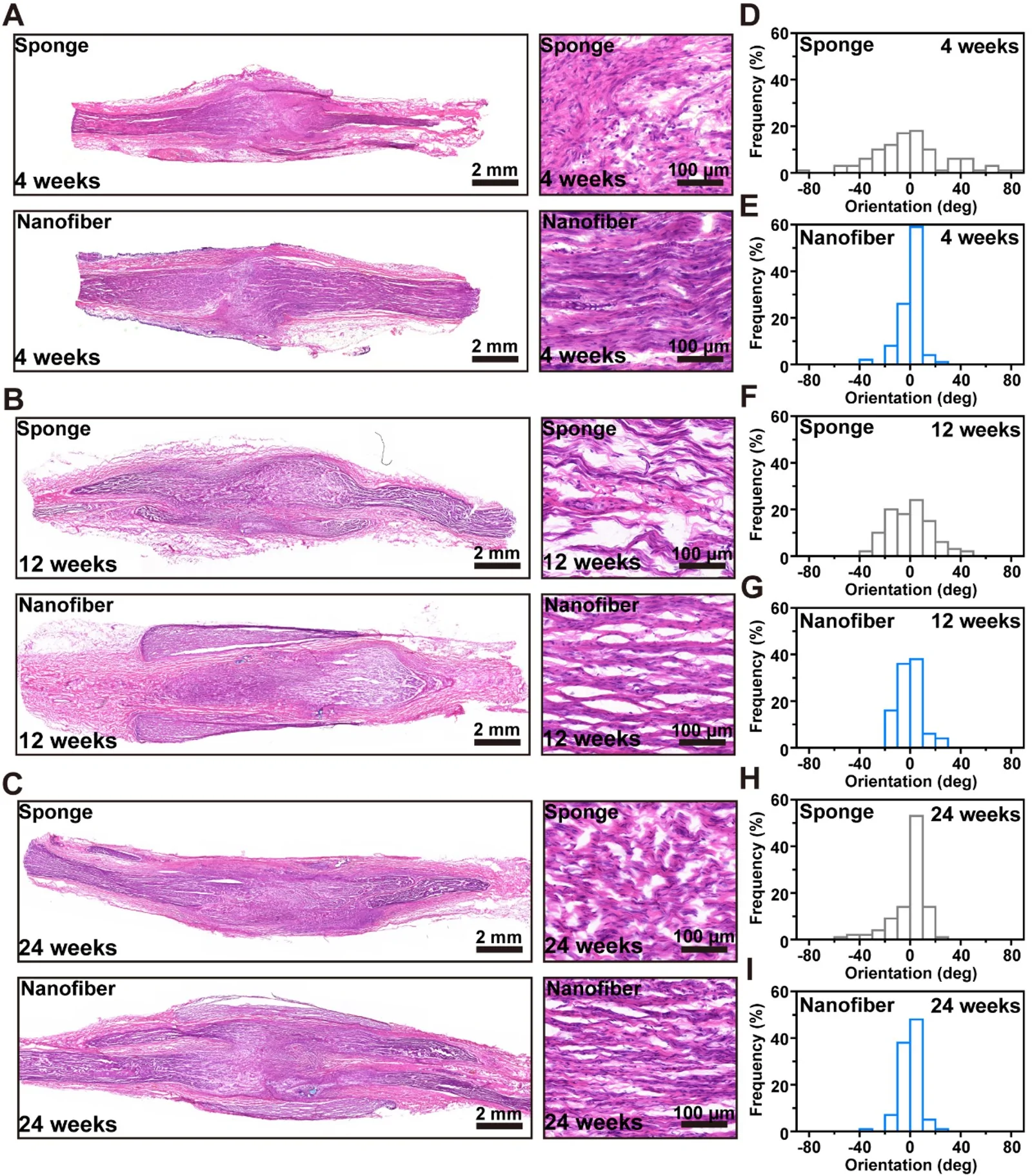
Bilayer nanofiber wrap promoted longitudinally organized nerve tissue regeneration during peripheral nerve repair. (A–C) Representative H&E staining images of regenerated nerve tissues in the commercial sponge wrap and bilayer nanofiber wrap groups at 4, 12 and 24 weeks post-surgery. High-magnification images were acquired from the neurorrhaphy region. (D–I) Orientation distribution analysis of regenerated nerve tissues in the sponge wrap and bilayer nanofiber wrap groups at 4, 12 and 24 weeks post-surgery.

At 12 weeks post-surgery, regenerated tissues in both the groups exhibited improved tissue continuity and progressively organized nerve-like architectures compared with the early regenerative stage (Figure 2B). The commercial sponge wrap group showed partial longitudinal remodeling of regenerated tissues, although heterogeneous regions and multidirectional fibrous arrangements could still be observed (Figure 2F). In contrast, regenerated tissues in the bilayer nanofiber wrap group appeared denser and more compact, with longitudinally aligned tissue bundles continuously extending along the regeneration axis (Figure 2G). Corresponding orientation analysis further demonstrated a more concentrated alignment profile in the bilayer nanofiber wrap group, supporting the presence of sustained aligned structural guidance during intermediate-stage tissue remodeling.

At 24 weeks post-surgery, regenerated tissues in both the groups exhibited further maturation and structural consolidation, with continuous nerve-like architectures observed throughout the repaired region, indicating substantial nerve tissue regeneration had been achieved (Figure 2C). Notably, tissues in the commercial sponge wrap group also showed partially restored longitudinal organization, suggesting the intrinsic capacity of peripheral nerves to undergo directional remodeling during regeneration. However, regenerated tissues within the bilayer nanofiber wrap group consistently maintained more compact and longitudinally oriented architectures, with aligned tissue bundles continuously extending along the repair axis even at the late regenerative stage. Orientation histogram analysis further confirmed the preservation of concentrated alignment profiles in the bilayer nanofiber wrap group during regeneration (Figure 2H and I). These findings indicate that the aligned nanofibrous architecture of the bilayer nerve wrap could continuously provide stable topographical guidance throughout post-neurorrhaphy regeneration, thereby supporting the formation of highly organized regenerated nerve tissues.

### Bilayer nanofiber wrap facilitated accelerated axonal regeneration and Schwann-cell-associated remodeling

Immunofluorescence staining of NF-200 and S100 was performed to evaluate axonal regeneration and Schwann-cell-associated neural remodeling within the repaired region. Progressive neural regeneration and structural maturation were observed in both the groups throughout the postoperative period, accompanied by increasingly continuous and organized nerve-like architectures over time. Representative immunofluorescence images from the neurorrhaphy region are shown in Figure 3, whereas continuous full-length longitudinal images spanning the proximal nerve segment, neurorrhaphy site and distal regenerated nerve segment are provided in Supplementary Figure S8.

**Figure 3:**
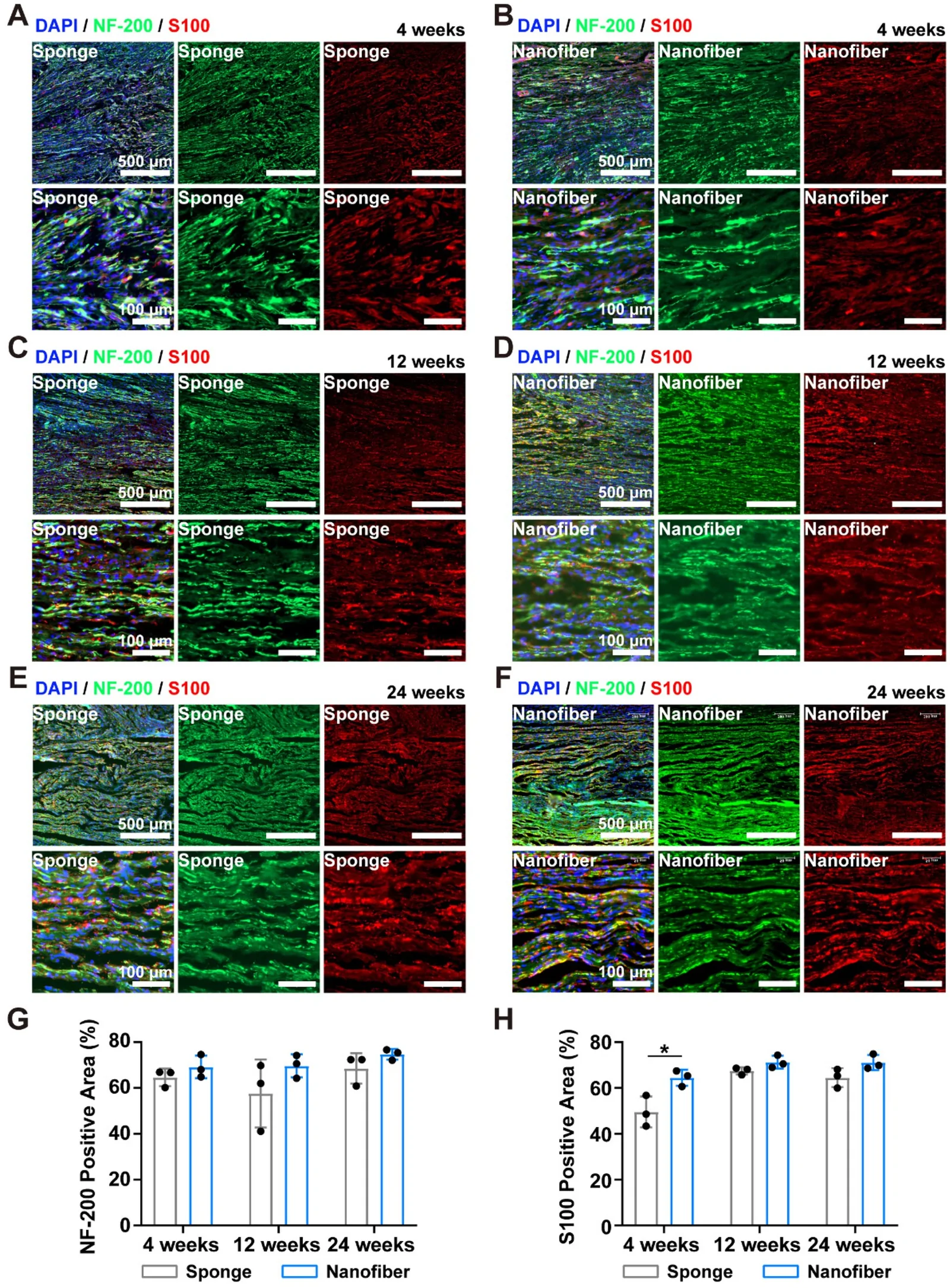
Bilayer nanofiber wrap facilitated aligned neural regeneration and Schwann-cell-associated remodeling. (A–F) Representative immunofluorescence staining images of NF-200, S100 and DAPI in regenerated nerve tissues from the commercial sponge wrap and bilayer nanofiber wrap groups at 4, 12, and 24 weeks post-surgery. Images were acquired from the neurorrhaphy region. Quantitative analysis of (G) NF-200-positive area and (H) S100-positive area at 4, 12 and 24 weeks post-surgery. Data are presented as mean ± SD (*n* = 3). *P < 0.05.

At 4 weeks post-surgery, evident NF-200-positive neural structures together with S100-positive Schwann-cell-associated tissues were observed in both the groups, indicating active neural regeneration within the repaired region (Figure 3A and B). Regenerated neural tissues gradually extended along the longitudinal axis, while S100-positive structures were distributed around regenerating axonal bundles, suggesting the initiation of coordinated neural remodeling. Notably, regenerated tissues in the bilayer nanofiber wrap group exhibited more continuous longitudinal neural bundle architectures together with denser and more aligned S100-positive distributions along the regeneration axis. Quantitative analysis further demonstrated significantly increased S100-positive areas in the bilayer nanofiber wrap group during the early regenerative stage (Figure 3H), indicating enhanced Schwann-cell-associated remodeling and accelerated reconstruction of the regenerative microenvironment. In parallel, NF-200-positive neural structures in the bilayer nanofiber wrap group appeared more continuously distributed across the neurorrhaphy region, suggesting accelerated early axonal regrowth under aligned topographical guidance.

At 12 weeks post-surgery, regenerated neural tissues in both the groups exhibited markedly improved continuity and structural organization compared with the early regenerative stage (Figure 3C and D). NF-200-positive neural structures became progressively denser and more continuous throughout the repaired region, while S100-positive tissues displayed increasingly organized distributions surrounding regenerated nerve bundles. Longitudinally arranged nerve-like architectures could be clearly observed in both the groups, indicating progressive maturation of regenerated neural tissues. Meanwhile, regenerated tissues within the bilayer nanofiber wrap group maintained more compact and aligned neural bundle organization together with continuously distributed Schwann-cell-associated structures extending along the regeneration axis. These findings suggest that the aligned nanofibrous architecture continuously promoted coordinated axonal extension and neural remodeling during the intermediate stage of peripheral nerve regeneration.

By 24 weeks post-surgery, both the groups exhibited further maturation and structural consolidation of regenerated neural tissues, characterized by highly continuous NF-200-positive nerve-like architectures throughout the repaired region (Figure 3E and F). Quantitative analysis demonstrated comparable NF-200-positive areas between groups during the late regenerative stage, indicating substantial axonal regeneration had been achieved in both the groups (Figure 3G). In parallel, S100-positive structures remained continuously distributed around regenerated neural bundles, reflecting stabilization of Schwann-cell-associated remodeling. Notably, regenerated tissues within the bilayer nanofiber wrap group still exhibited denser and more longitudinally organized neural architectures, suggesting that the aligned nanofibrous layer continuously supported organized axonal regeneration throughout peripheral nerve repair.

### Bilayer nanofiber wrap promoted accelerated remyelination and myelin maturation

Myelination and remyelination are critical hallmarks of functional peripheral nerve regeneration, as mature myelin sheaths are essential for rapid and stable electrical signal conduction. Early observations at 4 weeks post-surgery revealed that regenerated nerve tissues in both the groups remained at the initial stage of remyelination, characterized by sparse and immature myelinated fibers with relatively loose structural organization and incomplete myelin wrapping (Supplementary Figure S9). Nevertheless, regenerated tissues in the bilayer nanofiber wrap group already exhibited denser distributions of myelinated fibers together with more organized early-stage myelin architectures, indicating earlier progression of remyelination under aligned topographical guidance.

Toluidine blue staining was subsequently performed to evaluate the maturation of regenerated myelinated nerve tissues. At 12 weeks post-surgery, both the groups showed evident increases in myelinated nerve fiber formation accompanied by improved fascicular continuity and more compact nerve bundle organization (Figure 4A). Longitudinally arranged nerve-like structures together with progressively enriched myelinated fibers were clearly observed within regenerated tissues, reflecting active remyelination during the intermediate regenerative stage. Compared with the commercial sponge wrap group, regenerated tissues in the bilayer nanofiber wrap group displayed denser myelinated fibers and more continuous longitudinal fascicular architectures, suggesting enhanced myelin remodeling and accelerated nerve maturation. As regeneration progressed to 24 weeks, regenerated nerve tissues in both groups underwent further structural maturation characterized by markedly increased nerve fiber density, compact fascicular architectures and clearer nerve bundle organization (Figure 4B). Throughout regeneration, the bilayer nanofiber wrap group consistently maintained more organized and compact myelinated nerve structures.

**Figure 4:**
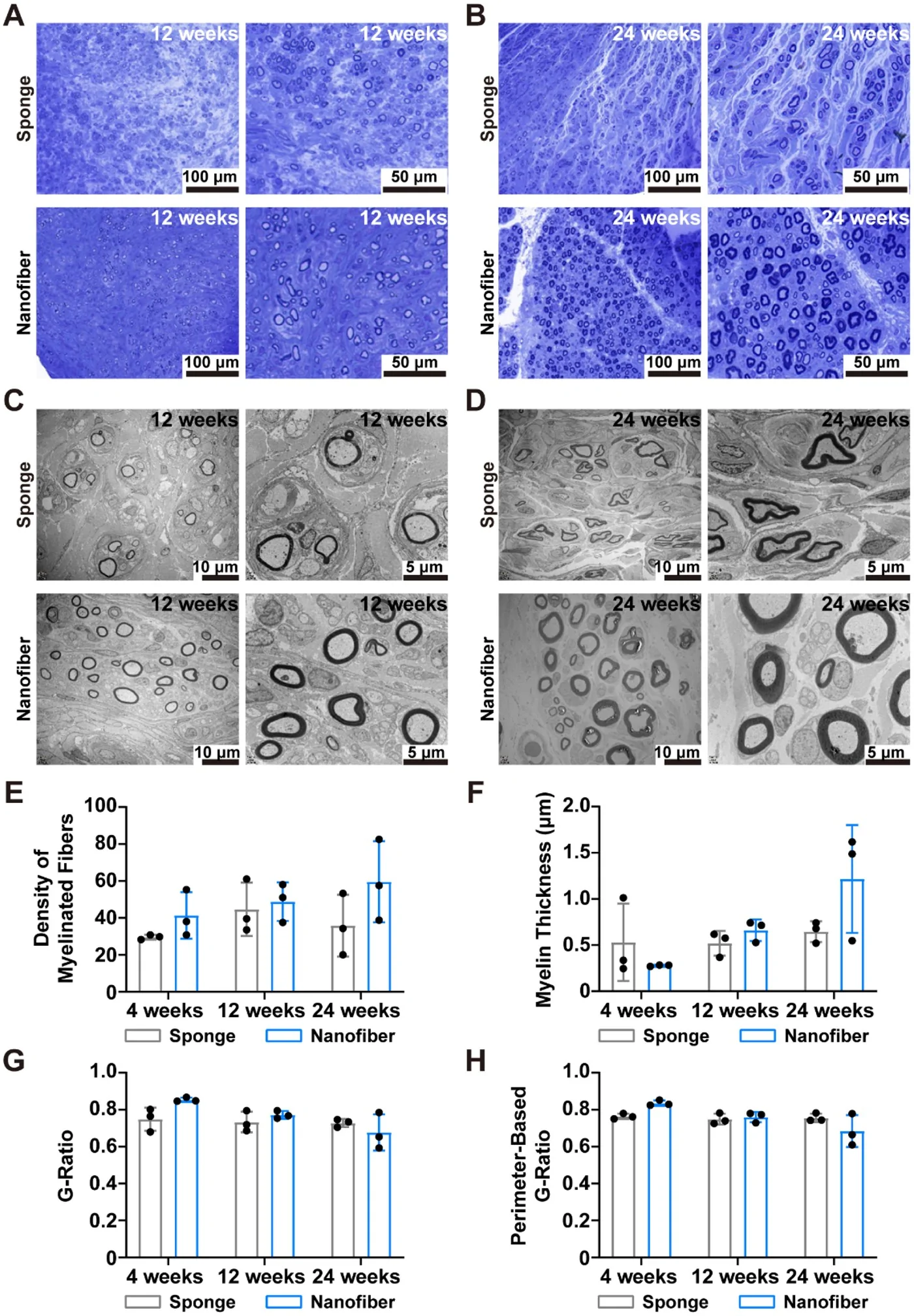
Bilayer nanofiber wrap promoted progressive remyelination and myelin ultrastructural maturation. (A, B) Representative toluidine blue staining images of regenerated nerve tissues in the commercial sponge wrap and bilayer nanofiber wrap groups at 12 and 24 weeks post-surgery. (C, D) Representative TEM images of regenerated nerve tissues in the commercial sponge wrap and bilayer nanofiber wrap groups at 12 and 24 weeks post-surgery. Quantitative analysis of (E) myelinated fiber density, (F) myelin thickness, (G) G-ratio and (H) perimeter-based G-ratio at 4, 12 and 24 weeks post-surgery. Data are presented as mean ± SD (*n* = 3).

TEM was further employed to investigate myelin ultrastructural maturation during regeneration. At 12 weeks post-surgery, regenerated tissues in both the groups displayed recognizable myelinated axons surrounded by multilayer myelin sheaths, indicating progressive restoration of axonal structural integrity (Figure 4C). Compared with the early regenerative stage, regenerated axons became denser and more regularly organized, while surrounding myelin sheaths exhibited increasingly compact lamellar structures. In particular, regenerated tissues in the bilayer nanofiber wrap group exhibited relatively thicker and more compact myelin sheath structures at this intermediate stage. By 24 weeks post-surgery, regenerated nerve fibers in both the groups underwent further ultrastructural maturation characterized by enlarged axonal morphology, compact multilamellar myelin wrapping and improved structural integrity (Figure 4D). Notably, regenerated tissues in the bilayer nanofiber wrap group continued to exhibit thicker and more compact myelin sheath organization, indicating sustained enhancement of myelin maturation.

Quantitative analysis further supported these histological and ultrastructural observations. The density of myelinated fibers progressively increased throughout regeneration, indicating continuous maturation of functional nerve fibers over time (Figure 4E). Myelin sheath thickness also gradually increased, reflecting improved myelin maturation and insulation capability during regeneration (Figure 4F). Meanwhile, both conventional *G*-ratio and perimeter-based *G*-ratio remained within relatively stable ranges throughout regeneration (Figure 4G and H), suggesting the establishment of physiologically appropriate axon–myelin relationships during nerve remodeling. Collectively, these findings demonstrated that neurorrhaphy-assisted nerve wrap implantation effectively supported progressive remyelination and maturation of regenerated nerve tissues, while the bilayer nanofiber wrap further enhanced remyelination quality and myelin maturation during peripheral nerve regeneration.

### Bilayer nanofiber wrap promoted electrophysiological reconstruction and functional nerve conduction recovery

Electrophysiological assessments were subsequently performed at 4, 12 and 24 weeks post-surgery to investigate functional neural reconstruction following peripheral nerve regeneration. Representative CMAP recordings revealed progressive recovery of electrophysiological signal conduction in both groups throughout the regenerative process (Figure 5A–C).

**Figure 5:**
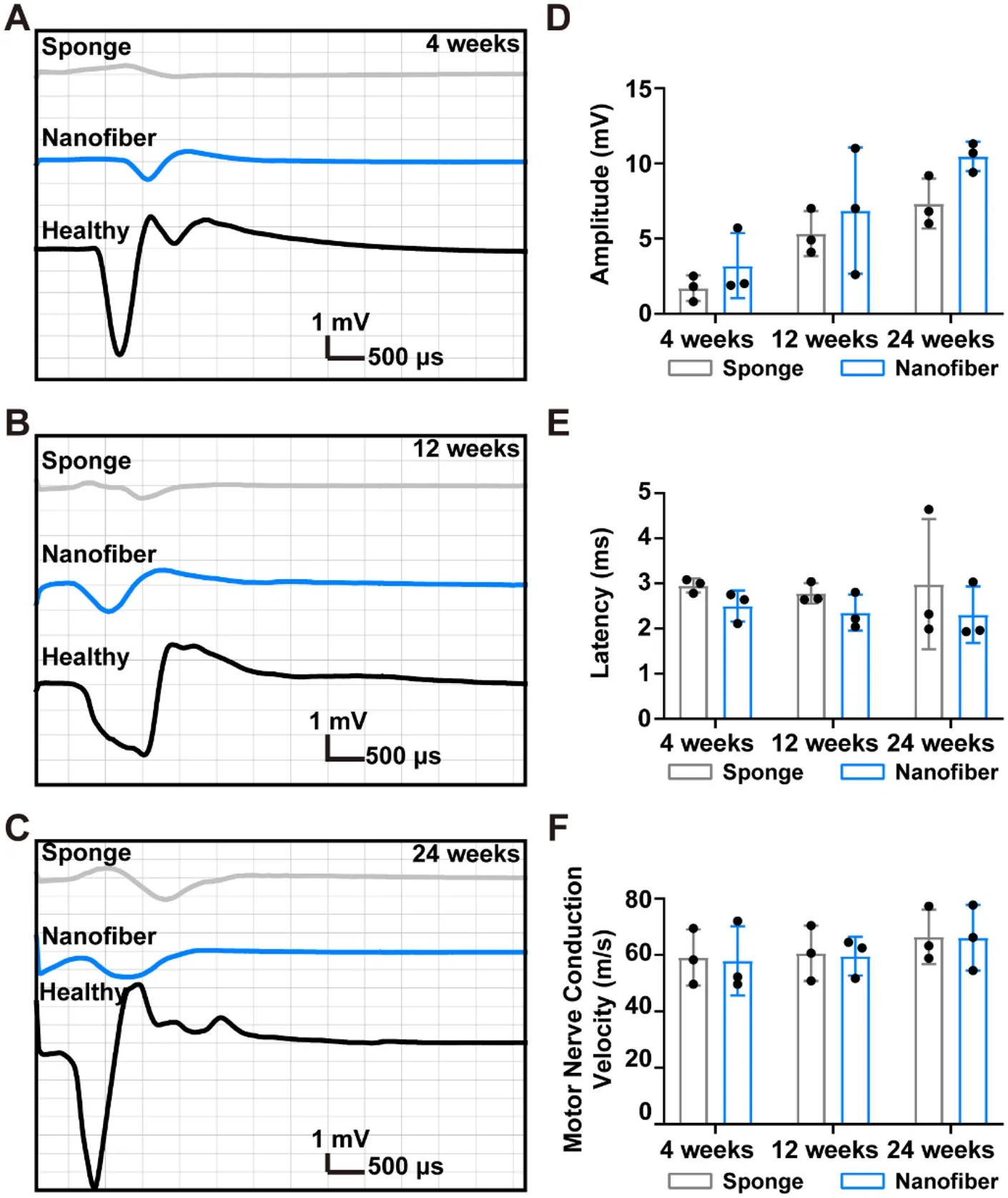
Bilayer nanofiber wrap facilitated electrophysiological reconstruction. (A–C) Representative CMAP waveforms recorded from the commercial sponge wrap, bilayer nanofiber wrap and healthy groups at 4, 12 and 24 weeks post-surgery. Quantitative analysis of (D) CMAP amplitude, (E) latency and (F) MNCV at 4, 12 and 24 weeks post-surgery. Data are presented as mean ± SD (*n* = 3).

At 4 weeks post-surgery, regenerated nerves in both groups exhibited relatively weak CMAP responses characterized by low-amplitude and broadened waveforms compared with healthy nerves, indicating the early stage of functional reinnervation. Notably, regenerated nerves in the bilayer nanofiber wrap group already exhibited relatively improved waveform continuity and more recognizable signal responses, suggesting accelerated early-stage neural conduction reconstruction. As regeneration progressed to 12 weeks, regenerated nerves in both the groups displayed increased CMAP amplitudes together with more recognizable biphasic waveform characteristics, improved signal continuity and clearer peak–trough morphology, suggesting progressive restoration of electrophysiological conduction function. Compared with the commercial sponge wrap group, regenerated nerves in the bilayer nanofiber wrap group exhibited relatively more complete waveform configurations with clearer peak morphology and improved waveform continuity, indicating enhanced synchronization of neural signal transmission during intermediate-stage regeneration. By 24 weeks post-surgery, electrophysiological waveforms underwent further maturation with enhanced waveform integrity, sharper peak morphology and more stable signal profiles. Regenerated nerves in the bilayer nanofiber wrap group consistently maintained more favorable waveform continuity and conduction characteristics, indicating sustained functional maturation of regenerated neural circuits.

Quantitative electrophysiological analysis further supported these waveform observations. CMAP amplitudes progressively increased during regeneration in both the groups, reflecting continuous recovery of functional axonal reconnection and muscle reinnervation capability (Figure 5D). Meanwhile, nerve conduction latency gradually decreased throughout the regenerative process, indicating progressive improvement in neural signal transmission efficiency (Figure 5E). In parallel, MNCV progressively increased from 4 to 24 weeks post-surgery in both the groups, demonstrating gradual maturation of regenerated nerve conduction function (Figure 5F). Notably, the bilayer nanofiber wrap group generally exhibited relatively improved electrophysiological conduction performance across multiple parameters during regeneration, consistent with accelerated axonal regeneration and remyelination observed histologically.

Collectively, these electrophysiological findings demonstrated that neurorrhaphy-assisted nerve wrap implantation effectively promoted functional neural reconstruction and progressive restoration of nerve conduction capability during peripheral nerve regeneration, while the bilayer nanofiber wrap further facilitated electrophysiological maturation and functional nerve conduction recovery.

### Bilayer nanofiber wrap supported functional locomotor recovery

Behavioral observations were performed at 24 weeks post-surgery to evaluate locomotor recovery after sciatic nerve repair. Representative images showed that animals in both the commercial sponge wrap group and the bilayer nanofiber wrap group achieved favorable functional recovery, with the operated hindlimbs actively participating in weight-bearing and stepping during locomotion (Figure 6A). Enlarged views of the affected paws further showed that the repaired limbs could contact the ground with relatively stable paw placement and restored plantar support, indicating effective recovery of limb loading and foot posture. These observations suggested that both wrap-assisted repair strategies supported substantial restoration of gross motor function by 24 weeks post-surgery.

**Figure 6:**
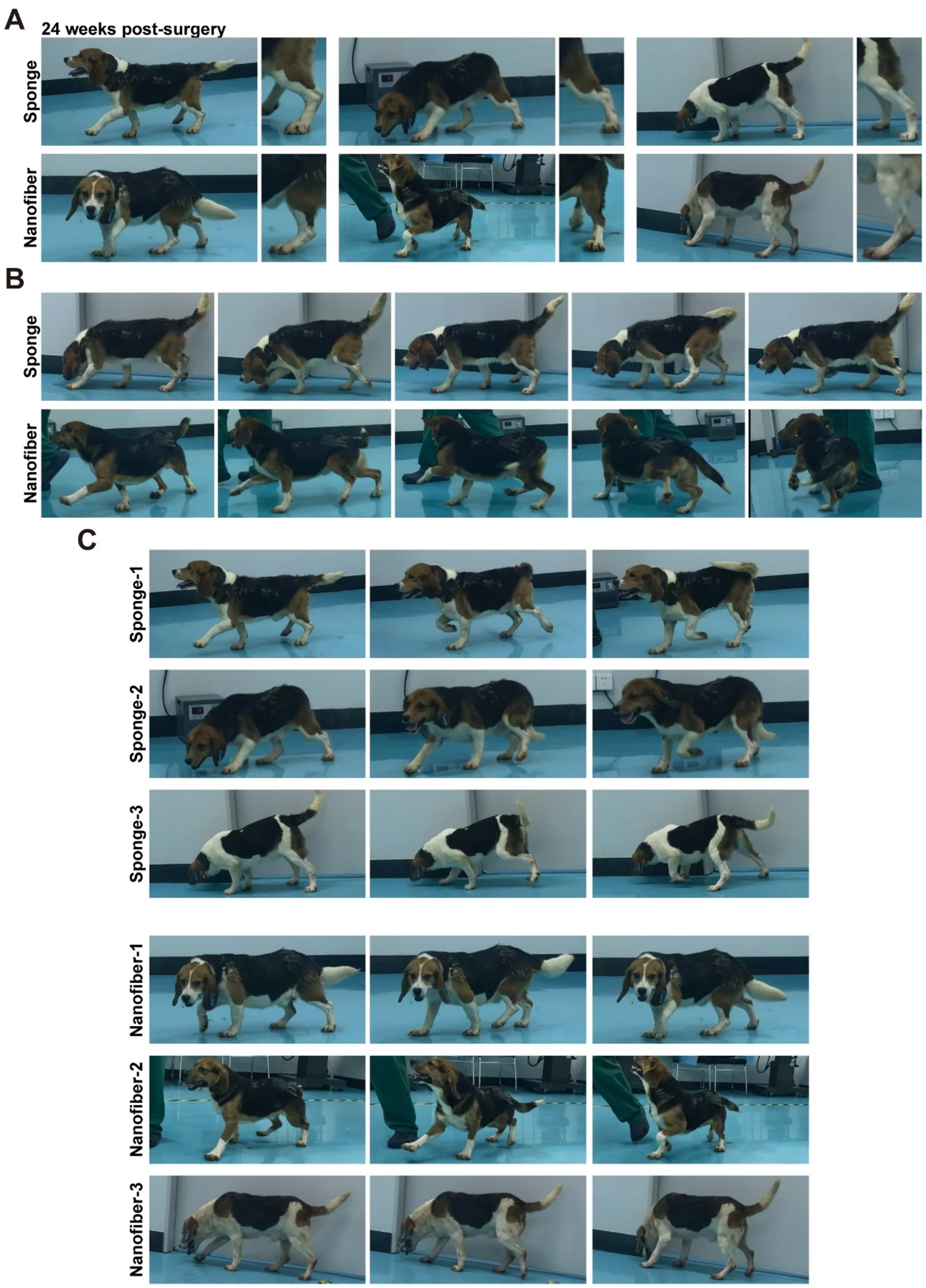
Bilayer nanofiber wrap promoted favorable locomotor functional recovery. (A) Representative locomotor images of the commercial sponge wrap and bilayer nanofiber wrap groups at 24 weeks post-surgery, showing hindlimb weight-bearing capability, paw placement and gait posture during walking. (B) Representative time-sequence gait images demonstrating continuous locomotor behaviors and hindlimb coordination in the commercial sponge wrap and bilayer nanofiber wrap groups at 24 weeks post-surgery. (C) Representative frame-by-frame gait analysis of the commercial sponge wrap and bilayer nanofiber wrap groups during hindlimb lifting, swinging and landing processes at 24 weeks post-surgery.

Time-sequence gait images further demonstrated coordinated walking behaviors in both the groups (Figure 6B). During continuous locomotion, animals were able to use the affected hindlimbs without obvious restriction, showing alternating limb movement, forward stepping and relatively stable body posture. The repaired limbs participated in the gait cycle with recognizable phases of limb lifting, forward swing and ground contact, suggesting recovery of coordinated neuromuscular control. Notably, several animals in the bilayer nanofiber wrap group displayed more active and dynamic locomotor behaviors, including relatively rapid stepping, stronger hindlimb push-off and occasional running- or jumping-like movements, indicating robust functional restoration after regeneration.

Frame-by-frame analysis of individual animals further supported the recovery of natural gait patterns (Figure 6C). Sequential images captured from representative walking cycles showed smooth transitions from paw lifting to forward swing and subsequent ground placement in both groups. Such coordinated movements were observed from different viewing angles, including lateral and posterior perspectives, further confirming that the repaired hindlimbs could be incorporated into the overall locomotor pattern. Consistently, supplementary behavioral videos showed that animals from both the groups achieved obvious locomotor recovery, while the bilayer nanofiber wrap group exhibited coordinated and vigorous limb movements during spontaneous walking (Supplementary Videos S2 and S3). Together with the electrophysiological findings, these behavioral observations indicated that wrap-assisted neurorrhaphy repair enabled meaningful functional recovery, and the bilayer nanofiber wrap supported the restoration of coordinated locomotor performance during peripheral nerve regeneration.

### Bilayer nanofiber wrap facilitated muscle restoration

The recovery of target skeletal muscle was subsequently evaluated to further investigate functional reinnervation after peripheral nerve repair. Gross observations of gastrocnemius muscles revealed progressive restoration of muscle morphology in both the groups throughout the regenerative process (Figure 7A). At 4 weeks post-surgery, varying degrees of muscle atrophy could still be observed in the operated limbs compared with the contralateral side. As regeneration progressed to 12 and 24 weeks, both groups exhibited gradual recovery of muscle volume and morphology, indicating progressive restoration of neuromuscular innervation. Notably, gastrocnemius muscles in the bilayer nanofiber wrap group displayed relatively fuller muscle morphology and improved structural preservation at the late regenerative stage. Quantitative analysis of the muscle wet weight ratio further supported the gross observations (Figure 7B). Both the groups showed progressive restoration of muscle mass during regeneration, and by 24 weeks post-surgery, the operated muscles recovered to a relatively favorable level, indicating substantial recovery of muscle maintenance capacity after reinnervation. The bilayer nanofiber wrap group exhibited relatively improved muscle wet weight preservation during regeneration.

**Figure 7:**
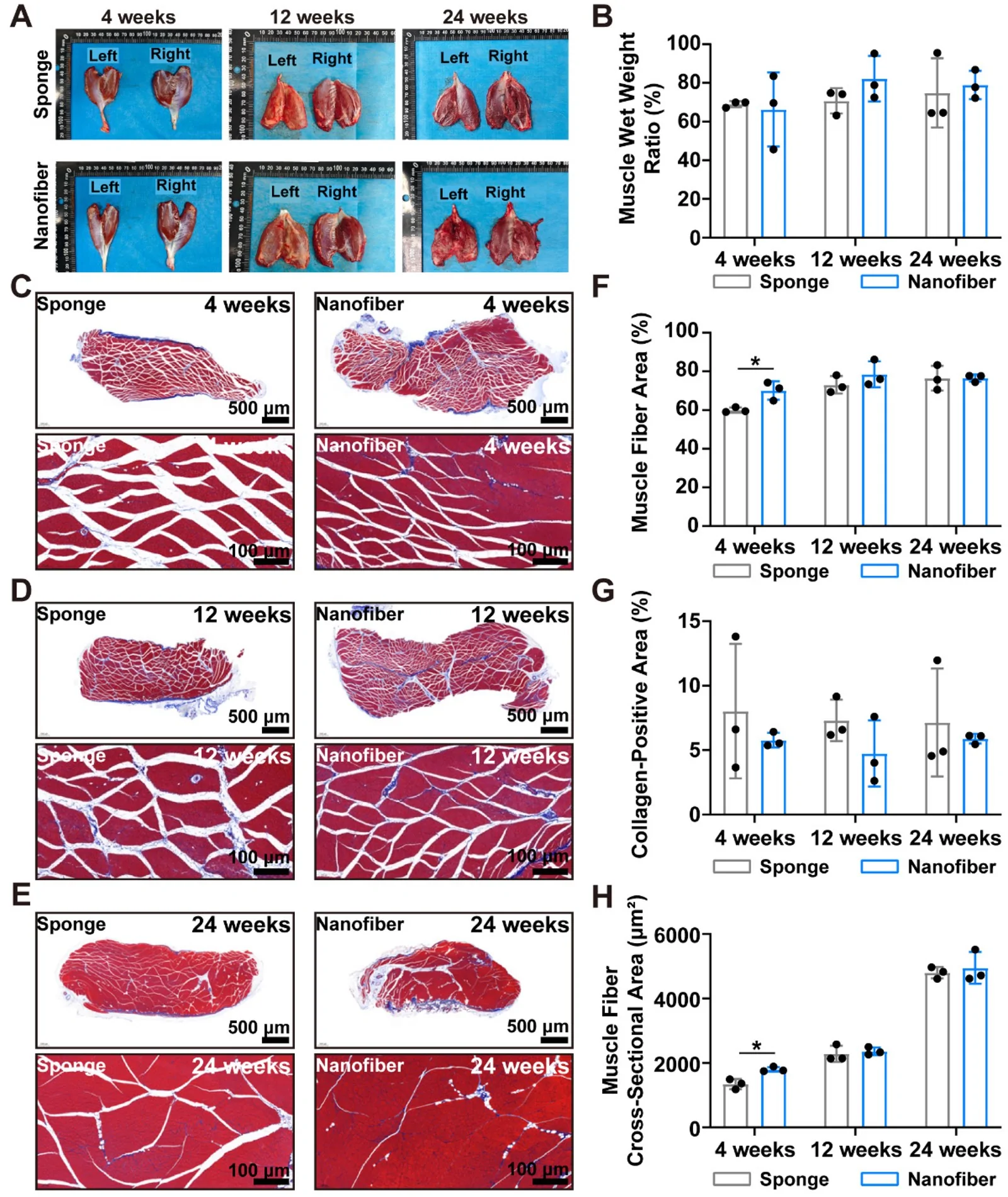
Bilayer nanofiber wrap promoted progressive muscle reinnervation and skeletal muscle recovery. (A) Representative gross images of gastrocnemius muscles in the commercial sponge wrap and bilayer nanofiber wrap groups at 4, 12 and 24 weeks post-surgery. (B) Quantitative analysis of muscle wet weight ratio at 4, 12 and 24 weeks post-surgery. (C–E) Representative Masson’s trichrome staining images of gastrocnemius muscle tissues in the commercial sponge wrap and bilayer nanofiber wrap groups at 4, 12 and 24 weeks post-surgery. Quantitative analysis of (F) muscle fiber area, (G) collagen-positive area and (H) muscle fiber cross-sectional area at 4, 12 and 24 weeks post-surgery. Data are presented as mean ± SD (*n* = 3). *P < 0.05.

Masson’s trichrome staining was subsequently performed to evaluate muscle fiber morphology and tissue remodeling. At 4 weeks post-surgery, regenerated muscles in both the groups exhibited relatively small and loosely arranged muscle fibers accompanied by enlarged interstitial spaces, reflecting early denervation-associated muscle atrophy (Figure 7C). Compared with the commercial sponge wrap group, the bilayer nanofiber wrap group displayed relatively larger muscle fibers with more compact fiber arrangement during the early regenerative stage. As regeneration progressed to 12 weeks, both the groups showed improved muscle fiber organization, together with enlarged muscle fiber morphology and reduced interstitial separation (Figure 7D). By 24 weeks post-surgery, regenerated muscles in both the groups exhibited substantial structural restoration characterized by densely packed muscle fibers and relatively mature muscular architectures (Figure 7E). Notably, muscles in the bilayer nanofiber wrap group displayed more intact and compact tissue morphology with well-preserved muscle bundle organization, indicating favorable muscle recovery following reinnervation.

Quantitative histological analysis further confirmed these observations. Muscle fiber area progressively increased throughout regeneration in both groups, while collagen-positive area remained relatively limited during the regenerative process (Figure 7F and G). In addition, muscle fiber cross-sectional area progressively recovered from 4 to 24 weeks post-surgery, indicating gradual restoration of muscle integrity and muscle trophic maintenance after nerve regeneration (Figure 7H). Consistent with the histological observations, the bilayer nanofiber wrap group exhibited relatively improved early-stage muscle fiber recovery together with favorable preservation of muscle morphology at the late regenerative stage. Collectively, these findings demonstrated that wrap-assisted neurorrhaphy repair effectively supported skeletal muscle restoration after PNI, while the bilayer nanofiber wrap facilitated favorable recovery of denervated target muscles.

## Discussion

A novel bilayer collagen nanofiber nerve wrap incorporating an inner aligned nanofibrous layer and an outer random fibrous layer was developed in the present study to establish a structurally guided regenerative microenvironment for peripheral nerve repair. Using a canine sciatic neurorrhaphy model, the regenerative performance and translational potential of this nerve wrap strategy were systematically investigated through histological, electrophysiological and functional evaluations. Peripheral nerve regeneration following severe nerve injury remains a prolonged and highly coordinated biological process involving axonal regrowth, Schwann-cell-associated remodeling, remyelination and target reinnervation [30]. However, conventional porous nerve wraps still face limitations in continuously guiding organized neural regeneration during tissue remodeling. In particular, insufficient topographical guidance within the regenerative microenvironment frequently results in heterogeneous fascicular organization, disordered axonal extension and incomplete electrophysiological reconstruction, ultimately limiting functional recovery. Therefore, developing a biomimetic nerve wrap capable of providing sustained aligned guidance has become an important strategy for peripheral nerve repair [31, 32]. Throughout the regenerative process, both the commercial sponge-like nerve wrap and the bilayer nanofibrous nerve wrap demonstrated favorable regenerative outcomes, including progressive neural tissue remodeling, electrophysiological restoration, locomotor recovery and target muscle reinnervation, indicating the effectiveness and translational feasibility of wrap-assisted neurorrhaphy repair. Importantly, the bilayer nanofiber wraps further promoted longitudinally organized neural regeneration, enhanced aligned neural fiber remodeling and accelerated earlier remyelination progression and functional recovery compared with the commercial sponge wrap. These findings suggest that aligned topographical guidance may represent an effective and clinically translatable design strategy for next-generation regenerative nerve wraps.

The favorable regenerative performance of the bilayer nanofiber wrap is closely associated with both its biomaterial composition and hierarchical structural design. Collagen has been widely recognized as one of the most clinically relevant biomaterials for peripheral nerve repair owing to its excellent biocompatibility, biodegradability, low immunogenicity and intrinsic extracellular matrix-mimicking bioactivity [33]. These characteristics not only provide collagen-based biomaterials with favorable biological activity for neural tissue remodeling but also facilitate their clinical translational potential [34]. Importantly, several collagen-based biomaterials have already been approved and applied in clinical regenerative medicine, further supporting the feasibility of collagen-derived nerve repair materials. Compared with conventional electrospinning strategies, blow-spinning technology also offers several advantages for nerve wrap fabrication, including higher production efficiency, improved scaffold permeability, reduced manufacturing complexity and enhanced scalability, all of which are important for future clinical translation and commercialization [25, 35].

The bilayer nanofiber nerve wrap developed in this study further integrates the functional advantages of nanofibrous architectures through a hierarchically organized structural design. First, the nanofibrous wrap exhibited favorable surgical handling capability during implantation and neurorrhaphy-assisted fixation. During peripheral nerve repair, repeated suturing between the wrap and epineurium is required to achieve stable fixation surrounding the coaptation site. Interestingly, the nanofibrous architecture demonstrated excellent tolerance toward needle penetration and surgical manipulation, effectively reducing the risk of wrap rupture during implantation. Unlike relatively brittle scaffold structures, the flexible nanofibrous network maintained structural integrity while adapting well to surrounding neural tissues and dynamic surgical environments. Consistently, intraoperative observations demonstrated that the bilayer nanofiber wraps preserved excellent integrity after repeated suturing throughout the neurorrhaphy-assisted wrapping procedure (Supplementary Figure S1), which may further facilitate surgical implantation and improve the practicality of wrap-assisted peripheral nerve repair in large-animal models. Second, the outer random fibrous layer primarily contributes to mechanical stability and structural protection throughout regeneration. The random fibrous organization provides continuous external support that helps maintain wrap integrity and resist collapse during tissue remodeling (Figure 1). Such protective effects are particularly important in neurorrhaphy-assisted repair models, where the nerve wrap not only functions as a regenerative microenvironment but also serves as a long-term protective covering structure surrounding the repaired nerve. Consistently, gross observations demonstrated that the bilayer nanofiber wraps maintained favorable structural continuity without obvious collapse or severe deformation throughout the 24-week regenerative period (Supplementary Figure S7), supporting stable neural tissue remodeling during repair. Third, the inner aligned nanofibrous layer establishes a continuous oriented topographical interface for regenerating neural tissues. The axon-mimicking aligned nanofibrous topology provides favorable cellular attachment interfaces together with directional regenerative guidance for neural remodeling. Histological observations further demonstrated that regenerated tissues within the bilayer nanofiber wraps continuously maintained highly longitudinally organized nerve-like architectures throughout regeneration. In particular, H&E staining revealed more concentrated orientation distributions and more continuous aligned neural bundle formation during early-stage regeneration (Figure 2), indicating that the aligned nanofibrous topology could provide sustained guidance for regenerating neural tissues. Through combining these structural characteristics, the bilayer nanofiber wraps simultaneously achieve surgical adaptability, mechanical stability, biological compatibility and topographical guidance capability, all of which are critical requirements for clinically translatable peripheral nerve repair materials.

Among the various structural factors involved in peripheral nerve repair, aligned topographical guidance represents one of the most important strategies for reconstructing organized neural regeneration [36, 37]. Native peripheral nerves possess highly ordered longitudinal architectures composed of microscale and nanoscale axonal bundles, myelinated fibers and aligned extracellular matrix components, all of which cooperatively support directional electrical signal conduction and functional target reinnervation. Therefore, biomaterials capable of recapitulating such anisotropic neural architectures have been extensively investigated in nerve tissue engineering and regenerative medicine. In the present study, the inner aligned nanofibrous layer of the bilayer nanofiber wraps was specifically designed to establish a biomimetic oriented regenerative interface capable of guiding neural regeneration across multiple hierarchical levels, including cellular regulation, tissue organization and functional reconstruction:

At the cellular level, aligned nanofibrous topographies regulate cellular morphology, directional migration and regenerative behaviors through mechanosensitive cell–matrix interactions. Such anisotropic architectures have been widely demonstrated to promote neuronal alignment, directional neurite extension and Schwann cell maturation, thereby providing essential structural cues for coordinated peripheral nerve regeneration [38–41]. In the context of the present zero-gap repair model, Schwann cells and regenerating axons remain highly responsive to local extracellular topographical cues at the neurorrhaphy interface. The aligned nanofibrous layer within the nerve wrap can therefore provide immediate contact-mediated guidance, which may activate mechanotransduction signaling pathways in both Schwann cells and axons [42–45]. This activation facilitates enhanced cellular alignment, neurite outgrowth and progressive maturation across the repair interface, even in the absence of a physical nerve gap. Importantly, such structurally mediated signaling is not strictly confined to the directly contacted region. Instead, it may propagate through coordinated cell–cell interactions and intercellular communication networks, leading to spatially coherent organization of regenerating neural tissues across the entire zero-gap repair environment [46–49]. In this manner, aligned topographical cues may contribute not only to local cellular orientation but also to higher-order tissue-level self-organization during early regenerative stages [50, 51]. Consistently, in our study, NF-200 and S100 immunofluorescence demonstrated progressive neural remodeling and Schwann cell-associated regeneration in both the experimental groups (Figure 3). Notably, the bilayer nanofiber wrap maintained a more longitudinally aligned neural architecture together with enhanced early-stage S100-positive remodeling, suggesting that aligned nanofibrous guidance may facilitate earlier Schwann cell activation and structured regenerative organization within the injury microenvironment. These findings are consistent with previous reports highlighting that aligned extracellular matrix architectures can regulate early nerve regeneration through contact-mediated mechanotransduction and glial–axonal coordination [52].

At the tissue level, the aligned nanofibrous interface further promoted structurally organized neural reconstruction throughout regeneration [53, 54]. H&E staining demonstrated that regenerated tissues within the bilayer nanofiber wraps maintained highly longitudinally organized nerve-like architectures across the entire regenerative process, exhibiting more continuous aligned neural bundle formation and concentrated orientation distributions during early-stage regeneration (Figure 2). Such aligned tissue remodeling was subsequently accompanied by accelerated and coordinated remyelination progression and neural maturation. Toluidine blue staining and TEM observations revealed progressively matured myelinated fibers together with compact multilamellar myelin formation in both the groups, while the bilayer nanofiber wraps visually demonstrated denser myelinated nerve fiber organization and relatively more mature myelin ultrastructure (Figure 4). These findings suggest that aligned nanofibrous guidance may facilitate more efficient axon extension, Schwann cell interaction and myelin formation by providing continuous physical guidance for regenerating neural tissues.

At the functional level, the structurally guided regenerative microenvironment further contributed to favorable electrophysiological reconstruction and behavioral recovery. Electrophysiological assessment demonstrated progressive restoration of neural signal conduction capability in both the groups throughout regeneration. Interestingly, the bilayer nanofiber wraps exhibited relatively improved early-stage waveform recovery together with shorter latency and more complete waveform morphology during regeneration, suggesting that aligned neural reconstruction may facilitate more efficient electrical signal transmission (Figure 5). We speculate that such effects may be associated with reduced axonal mismatch and more accurate target-oriented regenerative guidance provided by the aligned fibrous interface. During peripheral nerve regeneration, misdirected axonal regrowth frequently results in incomplete functional recovery despite structural regeneration. Therefore, physically guided aligned regeneration may help improve the accuracy of axonal extension and neural pathway reconstruction, thereby supporting more natural and coordinated functional restoration. Consistently, behavioral observations and muscle recovery analysis demonstrated favorable locomotor restoration, coordinated gait recovery and progressive muscle reinnervation in both the groups, while the bilayer nanofiber wraps further exhibited slightly improved trends in early-stage functional recovery and muscle remodeling (Figures 6 and 7). Collectively, these findings suggest that aligned nanofibrous topographical guidance can promote peripheral nerve regeneration across multiple hierarchical levels, ultimately facilitating more organized neural reconstruction and functional restoration.

Several limitations of the present study should also be acknowledged. First, although the current histological, electrophysiological and functional evaluations consistently demonstrated favorable regenerative outcomes, the overall experimental sample size remains relatively limited. Additional large-scale studies with more comprehensive quantitative analyses may further strengthen the reliability and reproducibility of the observed regenerative trends. Second, the present work primarily focused on neurorrhaphy-assisted peripheral nerve repair, which represents a relatively less challenging regenerative model compared with critical-sized nerve gap reconstruction. Although favorable regenerative outcomes were achieved under the current experimental conditions, more complex PNI models are still required before broader clinical translation can be fully established. In particular, large-gap nerve defect repair remains one of the major challenges in clinical peripheral nerve surgery, requiring prolonged directional axonal regeneration and stable regenerative support. Third, although a commercially available collagen nerve wrap was used as a clinically relevant comparator in the present study, the absence of a no-wrap control limits direct evaluation of the absolute contribution of nerve wrapping itself to nerve regeneration. Collectively, future studies incorporating both clinically established comparators and no-wrap controls in more clinically relevant gap-repair models with larger defect lengths will be necessary to further validate the therapeutic efficacy and clinical applicability of the present bilayer nanofibrous nerve wrap system. Investigations regarding critical-sized gap regeneration based on similar aligned regenerative strategies are currently being actively explored in our ongoing studies.

Despite these limitations, the present study still demonstrates important translational significance for clinically applicable peripheral nerve repair materials: firstly, compared with conventional small-animal models, the canine peripheral nerve model used in this work provides a more clinically relevant evaluation platform because of its closer anatomical dimensions, surgical procedures and functional rehabilitation characteristics relative to human peripheral nerves. Therefore, the favorable regenerative outcomes observed in this study further support the clinical applicability of the bilayer nanofiber nerve wrap design. These findings provide valuable large-animal preclinical evidence supporting the translational evaluation of clinically applicable nerve wraps. Future validation in more clinically relevant preclinical settings, together with long-term safety and functional assessments, will further optimize this design strategy and facilitate its eventual clinical translation. Collectively, the present canine model not only bridges the gap between conventional small-animal studies and clinical application but also provides a practical preclinical platform for evaluating next-generation nerve repair biomaterials before clinical use. Secondly, both the clinically available collagen nerve wrap and the bilayer nanofiber nerve wrap achieved favorable regenerative outcomes throughout the repair process, including neural tissue remodeling, electrophysiological recovery, locomotor restoration and muscle reinnervation, demonstrating the overall feasibility and effectiveness of wrap-assisted peripheral nerve repair in a clinically relevant large-animal model. In this context, the commercial nerve wrap serves as a clinically established benchmark for translational evaluation rather than a material-level comparator. Notably, the bilayer nanofiber nerve wrap showed consistent trends toward improved outcomes in neural tissue organization, Schwann cell-associated regeneration, remyelination progression and functional recovery. These results suggest that biomimetic topographical guidance may further enhance regeneration by promoting more structured nerve repair. These findings support the concept that integrating aligned nanofibrous architecture into clinically used nerve wraps represents a practical and translatable strategy for peripheral nerve repair. Thirdly, the translational significance of the present work is not limited to the specific nerve wrap itself but also lies in the validation of a clinically relevant design strategy centered on oriented nanofibrous guidance. The bilayer nanofiber nerve wrap simultaneously integrates surgical operability, structural stability, biomimetic topographical guidance and favorable regenerative capability within a relatively simple and scalable fabrication framework, thereby exhibiting promising potential for future clinical manufacturing and application. In particular, the aligned nanofibrous inner layer continuously provided directional regenerative guidance throughout remodeling while maintaining favorable tissue integration and wrap integrity, suggesting that topography-mediated regenerative modulation may remain effective throughout multiple stages of peripheral nerve repair.

Collectively, these results support the concept that biomimetic aligned nanofibrous architectures can serve as a clinically translatable regenerative strategy for next-generation peripheral nerve wraps and may provide valuable guidance for future translational studies in complex peripheral nerve reconstruction.

## Conclusion

In this study, we developed a bilayer collagen nanofiber nerve wrap via a blow-spinning strategy to establish a biomimetic regenerative microenvironment capable of providing sustained topographical guidance for peripheral nerve repair. The engineered wrap integrates an outer random fibrous layer that provides mechanical protection and surgical stability with an inner aligned nanofibrous layer that directs longitudinal neural regeneration. Using a canine sciatic neurorrhaphy model, the bilayer nanofiber wrap supported progressive neural tissue remodeling, aligned axonal regeneration, remyelination, electrophysiological reconstruction, locomotor restoration and target muscle reinnervation throughout repair. Compared with the commercially available nerve wrap, the bilayer nanofiber wraps further promoted longitudinally organized neural regeneration together with earlier remyelination and improved functional recovery, highlighting the importance of aligned topographical guidance during peripheral nerve regeneration. Collectively, these findings demonstrate that aligned nanofibrous guidance represents a clinically translatable design strategy for next-generation regenerative nerve wraps. Owing to its regenerative efficacy, surgical operability, scalable fabrication capability and translational potential demonstrated in a large-animal model, this bilayer collagen nanofiber wrap may serve as a promising platform for future clinical peripheral nerve reconstruction.

## Supplementary Material

rbag193_Supplementary_Data

## Data Availability

The data underlying this article are available in the article and in its online supplementary material.
