# Supplementary material for: Preclinical evaluation of a novel bilayer collagen nanofiber nerve wrap for accelerating axonal regrowth and myelination in a canine peripheral nerve injury model
Source: Regen Biomater. 2026 Sep 7;13:rbag193. doi: 10.1093/rb/rbag193 (PMC13661010; doi:10.1093/rb/rbag193)
Supplement: Supplementary file 1 [file rbag193_supplementary_data.zip › Supplementary_Data/Supplementary Data.docx]

**Supplementary Data**

**Preclinical Evaluation of a Novel Bilayer Collagen Nanofiber Nerve Wrap for Accelerating Axonal Regrowth and Myelination in a Canine Peripheral Nerve Injury Model**

*Kunkoo Kim^1,†^, Zhixin Wang^2,†^, Yaobin Yin^2^, Junlin Chen^1^, Pinyi Lai^1^, Jie Zhang^3^, Zide Wang^3^, Weitao Man^3^, Xiaobin Luo^3^, Yaosai Liu^1,4^, Yihsien Wu^5^, Haiyan Zhang^5^, Yongjia Zhang^5^, Chun-Yi Yang^5^, Jia Yang^1,6^, Zheng Cao^1,6,*^, Shanlin Chen^2,*^, Xiumei Wang^1,*^*

^1^State Key Laboratory of New Ceramic Materials, School of Materials Science and Engineering, Tsinghua University, Beijing 100084, China;

^2^Department of Hand Surgery, Beijing Jishuitan Hospital, Capital Medical University, Beijing 100035, China;

^3^Beijing Tsinghua Changgung Hospital, School of Clinical Medicine, Tsinghua University, Beijing 102218, China

^4^Department of Neurosurgery, Qilu Hospital (Qingdao), Cheeloo College of Medicine, Shandong University, Shandong, 266035, China

^5^Zhejiang Regenbiom Medical Technology Co., Ltd., Zhejiang 314501, China

^6^State Key Laboratory of New Ceramic Materials, Beijing Tsinghua Institute for Frontier Interdisciplinary Innovation, Beijing 102202, China

^*^ Correspondence address. E-mail: wxm@mail.tsinghua.edu.cn (X.W.), drcsl@qq.com (S.C.), 1988caozheng@163.com (Z.C.)

†: These authors contributed equally to this work.

**Table S1.** Primary and secondary antibodies

| **Antibodies** | **Vendor** | **Catalog number** | **Dilution (tissue)** | |
| --- | --- | --- | --- | --- |
| rabbit anti-S100 | Sigma | S2644 | 1:200 |  |
| mouse anti-NF-200 | Sigma | N0142 | 1:400 |  |
| Goat anti-Rabbit IgG (H+L) Secondary Antibody, Alexa Fluor 594 | Invitrogen | A-11012 | 1:1000 |  |
| Goat anti-Mouse IgG (H+L) Secondary Antibody, Alexa Fluor 647 | Invitrogen | A-21235 | 1:1000 |  |

**Figure S1.** Schematic illustration (A) and the representative surgical images (B) showing sciatic nerve exposure, sciatic nerve transection, and conduit-assisted wrapping of the neurorrhaphy site in the sponge nerve wrap and nanofiber nerve wrap groups.

**Figure S2.** Mechanical flexibility and structural recovery behavior of the bilayer nanofiber nerve wrap under external deformation.

**Figure S3.** Mechanical characterization of the bilayer nanofiber nerve wrap, including representative tensile stress–strain curve (A), quantitative analysis of tensile modulus (B), representative compressive stress–strain curve (C), and quantitative analysis of compressive modulus (D). Data are presented as mean ± SD (n = 3).

**Figure S4.** SEM images of the commercial sponge-like nerve wrap showing the isotropic porous microstructure at different magnifications (A) and cross-sectional SEM images of the sponge nerve wrap and nanofiber nerve wrap (B).

**Figure S5.** Quantitative analysis of white blood cell count (WBC) (A), red blood cell count (RBC) (B), neutrophil count (Neu) (C), and platelet count (PLT) (D) at 4, 12, and 24 weeks post-surgery. Data are presented as mean ± SD (n = 3).

**Figure S6.** Quantitative analysis of alanine aminotransferase (ALT) (A), blood urea nitrogen (BUN) (B), creatinine (CRE) (C), and creatine kinase (CK) (D) at 4, 12, and 24 weeks post-surgery. Data are presented as mean ± SD (n = 3).

**Figure S7.** Representative gross images of regenerated nerve tissues in the sponge nerve wrap and nanofiber nerve wrap groups at 4, 12, and 24 weeks post-surgery.

**Figure S8.** Representative immunofluorescence staining images of NF-200 (green), S100 (red), and DAPI (blue) in regenerated nerve tissues from the commercial sponge wrap and bilayer nanofiber wrap groups at 4, 12, and 24 weeks post-surgery. Images cover the continuous longitudinal regenerated nerve segment, including the proximal nerve segment, neurorrhaphy site, and distal regenerated nerve.

**Figure S9.** Representative toluidine blue staining images (A) and transmission electron microscopy (TEM) images (B) of regenerated nerve tissues in the sponge nerve wrap and nanofiber nerve wrap groups at 4 weeks post-surgery.
